# An Interesting Case

**Published:** 1881-10

**Authors:** 


### AN INTERESTING CASE.

About four years ago, a woman was sent to me by her physician, complaining of very intense pain in her ears and teeth, and desiring me to extract a sound upper molar, but I demurred and removed an adjacent root. She returned in a few days, having experienced no relief and insisted on the removal of the tooth designated. I reluctantly complied with her request. When I saw her a few days afterward, she told me that all pain had ceased, but this respite was of short duration, for in a few weeks she returned as bad as ever and wanted another sound tooth extracted, I removed it, and again the pain disappeared to again reappear. She came to me every eight or ten weeks to get a sound tooth extracted. At last, as my conscience would not allow me to continue this practice, I sent her to Dr. Von Langsdorf. He removed two roots without giving her any relief. I drilled into the ailing tooth and removed the pulp, and the pain ceased again. A tooth removed sometime afterward was examined by the attending physician, and the pulp found to contain particles of ossific matter.

The patient now became so weak that she was confined to her bed, and her physician would not consent to any further operations, fearing they would not be beneficial, so she called in a surgeon who removed from time to time, fourteen teeth. She then presented herself to me with only three upper incisors remaining, and as she insisted so strongly, I extracted them.

For six days following the operation she suffered such severe pain, and her conduct was such, that her relatives believed she was insane. The pain gradually subsided, and to-day she wears a full upper and lower set of teeth, and has fully recovered her health.

Sometime before this I had a similar case. It was a servant girl for whom I, after entering the pulp cavity, cauterizing, removing the pulp, etc., etc., extracted several teeth. She then went into service at Basel, and I lost sight of her. I saw her about a year ago, she had suffered with severe pain till all her teeth were removed.

The cause in both these cases was ossification of the pulp. (*Dr. Von Muhlhausler, Lahr in Vierteljahresschrift des Vereins Deutcher Zahnkunstler for August*, 1881.)

